# A review on charred traditional Chinese herbs: carbonization to yield a haemostatic effect

**DOI:** 10.1080/13880209.2019.1645700

**Published:** 2019-08-10

**Authors:** Zhi Chen, Si-Yong Ye, Ying Yang, Zhong-Yuan Li

**Affiliations:** a College of Pharmacy, Shandong University of TCM, Jinan, China;; b Department of Pharmacy, Jinan Second People's Hospital, Jinan, China

**Keywords:** Charcoal drug, carbon dots, traditional Chinese medicine

## Abstract

**Context:** Charcoal of Chinese drugs is a kind of special processing product in Chinese medicine and used for treatment of haemoptysis, hematemesis and haemorrhage in the clinic during ancient times. During carbonizing, significant changes occur in chemical constituents and the efficacy of haemostasis will be enhanced. But the quality control standard of ‘carbonizing retains characteristics’ should be followed.

**Objective:** This review introduces the typical methods of carbonizing, which highlight current research progress on haemostatic substances of charcoal drugs so as to provide a reasonable explanation for the theory of haemostasis treated by charcoal medicine.

**Methods:** English and Chinese literature from 2004 to 2019 was collected from databases including Web of Science, PubMed, Elsevier and CNKI (Chinese). Charcoal drug, chemical constituents, processing, haemostasis and carbon dots were used as the key words.

**Results:** Charcoal drugs mainly play a haemostatic role and the effect can be classified into four types to stop bleeding: removing blood stasis, cooling blood, warming meridians and astringing. Changes in composition lead to changes in pharmacodynamics. Carbonizing methods and basic research on haemostasis material in charcoal drugs have also been summarized.

**Conclusions:** This review summarizes the classification of charcoal drugs and highlights the possible material bases for the haemostatic effect of charcoal drugs in recent years, providing new insights to future research.

## Introduction

During processing, significant changes in chemical profiles occur, so it is essential to ensure the safety and quality of TCMs. The Chinese medicinal materials originate from plants, animals or minerals and must be processed before they can be used in clinics, and this is one of the characteristics of traditional Chinese medicine (Sheridan et al. 2015). In contrast, only few processing methods are recorded in the pharmacopoeias of other countries (Guo et al. 2015). There are many ways to process, including: stir-frying, stir-frying without excipients, stir-frying with liquid excipients, stir-frying with solid excipients, steaming, boiling, stewing, etc. (Wu, Wang et al. 2018). Through accumulated experience and technology development, a special theory of haemostatic effect by charcoal TCM targeting haemorrhagic diseases has been formed.

Haemostasis by charcoal TCM dates back to the Han Dynasty and has a history of more than 2000 years. It has played an important role in TCM throughout history, which gradually developed into a special theory of haemostasis by charcoal TCM targeting haemorrhagic diseases. In Treatise on Febrile and Miscellaneous Diseases, Zhang Zhongjing first put forward the standard of charcoal TCM, ‘medicine should be burned as charcoal with function preserved’ (Yan 2012). Since then, the standard has been inherited untill this day. Modern medical researchers have indicated that the haemostatic function of some herbs could be produced after carbonizing (Kong et al. 2011; Li, Kong et al. 2011). Research has shown that raw *Rubiae Radix et Rhizoma* has the effect of removing blood stasis and haemostasis, while carbonized *Rubiae Radix et Rhizoma* mainly has a haemostatic effect. This further demonstrates the traditional processing theory of ‘promoting blood circulation with crude herbs and stopping bleeding with processed herbs’ (Shan et al. 2014).

**Figure 1.**
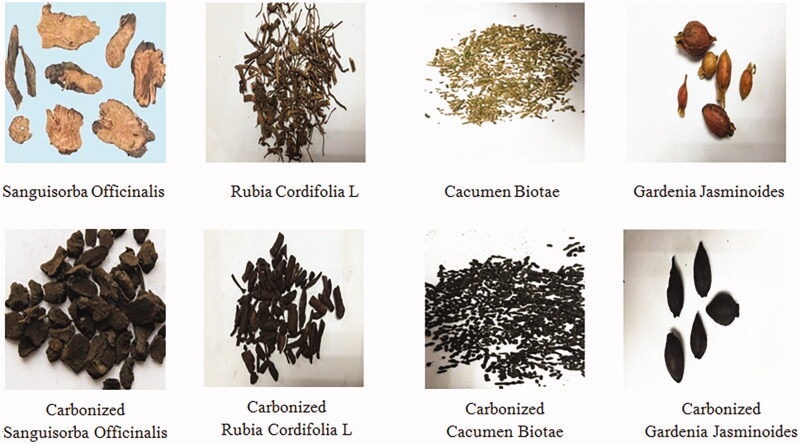
Four decoction pieces recorded as well as their respective carbonized products.

Four types of common Chinese charcoal medicines are shown in Figure 1. Partial carbonization is a commonly used method in the processing of some traditional Chinese herbs, where the drugs are carbonized to increase carbon and tannin or to generate new compounds to improve the haemostatic effect. Though classic carbonizing theory and methods have been proven reliable and reasonable in the long-standing clinical practice of TCM, the underlying scientific reason remains largely unknown. Modern scholars hold that the standard allows medicine to retain its partial properties and play a role in stopping bleeding at the same time. Though there are many processing methods of charcoal drugs, there are no unified objective quantitative indexes for standard of processing of charcoal drugs due to restrictions of objective factors, such as technology and conditions that severely restricts the development of TCM (Zhao et al. 2010).

A charring degree of medicine during processing is evaluated according to its internal and external colours, but the material basis and the function mechanism are still important and difficult issues in modern research (Fei et al. 2018). In most cases, the structures and contents of constituents may be altered simultaneously, and that may lead to the changed pharmacological activity of a certain TCM. The recent study on the material basis of the haemostatic effect of charcoal drugs still focus on changes of active ingredients of drugs after processing and its basic chemical mechanism with lots of related literature reports. A systematic review satisfies an urgency need to summarized the accumulated knowledge of the past few decades. Herein, we comprehensively summarized research literature on chemical constituents, pharmaceutical effect, different types of charcoal drugs and highlighted the possible material bases for the haemostatic effect of charcoal drugs.

## Classification and efficacy of charcoal drugs

**Figure 2.**
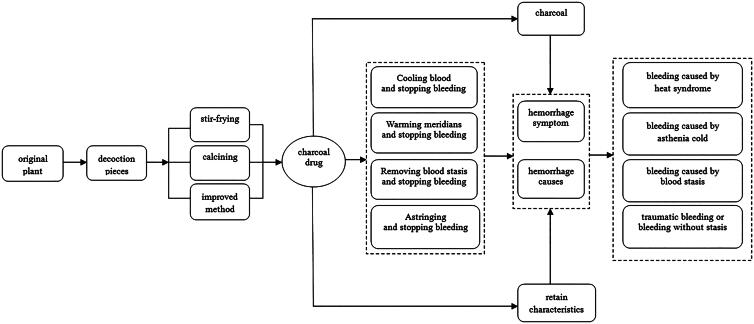
Four types of charcoal drugs and their effective pathway of hemostasia mechanism.

At present, Chinese Pharmacopeia (2015 edition) includes 25 types of charcoal drugs, such as carbonized *Herba Cirsii Japonici* (Dajitan), carbonized hair (Xueyutan) and carbonized *Rheum Officinale* (Dahuangtan) (Chinese Pharmacopoeia Commission 2015). However, more than 70 types of charcoal drugs were widely used in the clinic and far more than those are included in Chinese Pharmacopeia. By carbonizing, traditional Chinese medicine can change its property and enhance its effect, especially haemostatic effect. Due to its long history and rich content, it reflects the simple dialectical thoughts of traditional Chinese culture (Ma et al. 2017). As the standard of ‘carbonizing retains characteristics’ is followed, some original properties and effects of haemostatic drugs are maintained after being carbonized. According to properties and efficacy of charcoal drugs, the effect can be classified into four types to stop bleeding such as: removing blood stasis, cooling blood, warming meridians and astringing (Figure 2).

### Type 1: charcoal drugs for removing blood stasis and stopping bleeding

Common clinical charcoal drugs for removing blood stasis and stopping bleeding mainly include carbonized *Cortex Moutan* (Danpitan), carbonized *Pollen Typhae* (Puhuangtan), carbonized *Selaginella* (Juanbaitan) and carbonized *Radix Rubiae* (Qiancaotan), etc. Raw *Pollen Typhae* is mainly used for promoting blood circulation and stopping pain, while charred *Pollen Typhae* is of unsmooth nature and mainly used for stopping bleeding (Shen et al. 2015). Lotus leaf is a widely used traditional Chinese medicine and it also can be used as graphitic N-dominated porous carbon catalyst and solar water evaporation devices (Wang, Xiang et al. 2018; Liao, Chen et al. 2019). Raw lotus leaves are characterized by relieving summer-heat and clearing heat and sending up the lucid *yang*, while carbonized lotus leaves show strong effects of inducing astringency, removing blood stasis and stopping bleeding (Peng et al. 2013).

### Type 2: charcoal drugs for cooling blood and stopping bleeding

Such drugs refer to medicines cold in nature. They can restore over speed blood circulation caused by heat thus to avoid bleeding. They are mainly applied to treatment of bleeding symptoms caused by exuberant fire and heat, fire excess from yin deficiency, superficial venules and lymph vessels and forced exuberant blood circulation. Common drugs for cooling blood and stopping bleeding include carbonized *Cirsium Japonicum* DC (Dajitan), carbonized *Cirsium Setosum MB* (Xiaojitan), carbonized *Sophora Japonica* (Huaihuatan), carbonized *Sanguisorba Root* (Diyutan) and carbonized *Radix Scutellariae* (Huangqintan), etc.

### Type 3: charcoal drugs for warming meridians and stopping bleeding

These medicines can be applied to deficiency-cold haemorrhage syndromes caused by spleen failing to manage blood and unstable Chong Channel, such as hematochezia, uterine bleeding, purpura and dark tint face. This type of medicine is mild in nature with weak stypticity and can be used as interior-warming drugs alone. After carbonizing, TCM can strengthen its warm nature and reduce its pungent and dispersing nature. For example, raw *Artemisia Argyi* is used for cooling blood and stopping bleeding with cold nature. After processing, charcoal *Artemisia Argyi* is used for warming meridians and stopping bleeding with warm nature and often applied to treatment of insufficiency-cold female bellyache, uterine bleeding, dysmenorrhea and postpartum abdominal pain, etc. (Chen et al. 2016). After carbonizing, radix ginger strengthens its warm nature and reduces its pungent and dispersing nature. It can be used for warming the middle energizer and stopping diarrhoea, warming meridians and stopping bleeding and treating insufficiency-cold uterine bleeding, hematochezia and stomach ache (Mo et al. 2015).

### Type 4: charcoal drugs for astringing and stopping bleeding

Charcoal drugs for astringing and stopping bleeding are used for patients with endless massive bleeding. It is mainly used to treat functional uterine bleeding, induced abortion bleeding and other external causes of gynaecological bleeding. During treatment, extravasated blood caused by bleeding stopping should be avoided. Common drugs for astringing and stopping bleeding include carbonized palm (Zonglvtan), carbonized *Nodus nelumbinis Rhizomatis* (Oujietan) and carbonized hair (Xueyutan), etc.

Though great changes of properties and efficacy of traditional Chinese medicine may occur before or after carbonizing, ‘property maintenance’ is a basic principle for carbonizing. *Sanguisorba officinalis* root is tenacious. Standard charred *Sanguisorba officinalis* root should be internally reddish brown and externally burned black. In other words, it is constituted by a charry part and a part maintaining its property. As a result, in addition to be used for stopping bleeding, different charcoal drugs have different clinical targets. For instance, charcoal drugs for removing blood stasis and stopping bleeding are mainly applied to treatment of bleeding symptoms caused by poor blood circulation. Therefore, we should study charcoal drugs on the basis of drug properties and efficacy and clinical applications. On the principle of treatment based on syndrome differentiation of TCM, we should discuss mechanisms of bleeding stopping and processing of charcoal drugs in combination with overall animal models by adopting modern means and methods with multiple approaches and indexes. Twenty-five carbonized herbs recorded in Chinese Pharmacopeia (2015 edition) are described below and listed in Table 1.

**Table 1.** Classification of haemostatic agents and representative carbonized Chinese herbal medicine listed in CP (2015 edition).

Classification	Symptoms	Raw drug	Charred drug	Processing method	Comment
Cooling blood and stopping bleeding	Blood-heat bleeding	*Cirsii Japonici Herba*	*Cirsii Japonici Herba* Carbonisata	Stir-frying until the surface is black	Fry charcoal^a^
		*Cirsii Herba*	Carbonized *Cirsii Herb*	Stir-frying until the surface is black	Fry charcoal
		*Sanguisorbae Radix*	Carbonized *Sanguisorbae Radix*	Stir-frying until carbonized (black outside and charred inside)	Fry charcoal
		*Sophorae Flos*	Carbonized *Sophorae Flos*	Stir-frying until the surface is ustulate	Fry charcoal
		*Rhei Radix Rhizoma*	Carbonized *Rhei Radix Rhizoma*	Stir-frying until carbonized (black outside and charred inside) with burnt flavour	Fry charcoal
		*Imperatae Rhizoma*	Carbonized *Imperatae Rhizoma*	Frying until the surface is ustulate	Fry charcoal
		*Platycladi Cacumen*	Carbonized *Platycladi Cacumen*	Stir-frying until carbonized (black outside and charred inside)	Fry charcoal
		*Junci Medulla*	Carbonized *Junci Medulla*	Calcining to black	Calcining
		*Phellodendri Chinesis Cortex*	Carbonized *Phellodendri Chinesis Cortex*	Stir-frying until the surface is black	Fry charcoal
		*Phellodendri Amurensis Cortex*	Carbonized *Phellodendri Amurensis Cortex*	Stir-frying until the surface is black	Fry charcoal
		*Dryopteridis Crassirhizomatis Rhizoma*	*Dryopteridis Crassirhizomatis Rhizoma* Carbonisatum	Stir-frying until the surface is ustulate	Fry charcoal^a^
Warming meridians and stopping bleeding	Cold bleeding	*Zingiberis Rhizoma*	Carbonized *Zingiberis Rhizoma*	Stir-frying until carbonized (black outside and charred inside)	Fry charcoal
		*Artemisiae Argyi Folium*	Carbonied *Artemisiae Argyi Folium*	Stir-frying Artemisiae Argyi Folium until the surface is black, spray vinegar and stir-fry until dry	Fry charcoal
Removing blood stasis and stopping bleeding	Congestion bleeding	*Selaginellae Herba*	Carbonized *Selaginellae herba*	Stir-frying until the surface is black	Fry charcoal
		*Rubiae Radix et Rhizoma*	Carbonized *Rubiae Radix et Rhizoma*	Stir-frying until the surface is black	Fry charcoal
		*Nelumbinis Receptaculum*	Carbonized *Nelumbinis Receptaculum*	Calcining to black	Calcining
		*Nelumbinis Folium*	Carbonized *Nelumbinis Folium*	Calcining to black	Calcining
Astringing and stopping bleeding	Massive haemorrhage	*Mume Fructus*	*Mume Fructus* carbonisatum	Stir-frying until the surface is black and bump	Fry charcoal
		*Granati Pericarpium*	Carbonized *Granati Pericarpium*	Stir-fry until dark yellow on the surface and brown on the inside	Fry charcoal
		Crinis (non-medicine)	Crinis Carbonisatus	Take hair, remove impurities, wash off oil and dirt by alkaline, calcining to black	Calcining^a^
		*Schizonepetae Herba*	*Schizonepetae Herba* Carbonisata	Stir-frying until carbonized (black outside and charred inside)	Fry charcoal^a^
		*Schizonepetae Spica*	*Schizonepetae Spica* Carbonisata	Stir-frying until carbonized (black outside and charred inside)	Fry charcoal^a^
		*Typhae pollen*	Carbonized *Typhae pollen*	Frying until the surface is brown	Fry charcoal
		*Trachycarpi Petiolus*	Carbonized T*rachycarpi Petiolus*	Calcining until the surface is black and luster, interior is burnt yellow and fibrous	Calcining
		*Nelumbinis Rhizomatis Nodus*	Carbonized *Nelumbinis Rhizomatis Nodus*	Frying until the surface is black and the interior is tan	Fry charcoal

The above processing methods are from Processing Principles of the Chinese Pharmacopeia Appendix II D.

^a^ Is to point to independent quality standards.

## Carbonizing methods

### Traditional methods

The primary method to prepare charcoal medicine is carbonizing the medicine by high-temperature heating (Liu et al. 2018). Through carbonizing, some of the chemical components in drugs will be changed (Sun et al. 2013). According to the TCM theory, some of the components will be lost during the processing, while the efficacy of haemostasis will be enhanced and the toxic effect will be reduced (Chai et al. 2012). Two primary traditional methods of carbonizing are: carbonizing by stir-frying and carbonizing by calcining (also known as wok-covering calcining). Both methods are suitable for general drugs. Carbonizing by stir-frying means to heat medicine with a preheating vessel of high or moderate heat until the medicine turns into internally reddish brown and externally burned black. It belongs to plain-frying. Its main purpose is to endow medicine with an effect of stopping bleeding or strengthen the effect of stopping bleeding. It can be applied to stir-frying of rhizome drug, such as carbonized *Rheum officinale*, carbonized *Zingiberis Rhizoma* and carbonized *Cortex Moutan*, etc. Carbonizing by calcining means to heat and carbonize medicine in high-temperature and anoxic condition. It is appropriate for loose or light medicines that can be carbonized easily (such as hair and *Juncus effusus*) and solid medicines that cannot be stir-fried easily (such as *Radix Rehmanniae* and *Nodus Nelumbinis Rhizomatis*). It is difficult to control the stir-frying degree of charcoal drugs by these traditional methods of carbonizing, for these methods have many defects. Uneven heating of medicine can cause ashing, carbonization or raw pieces. Due to light weight of leaf medicine, it is difficult to control duration and heating degree, leading to a high rate of waste. As the interior of root medicine is not dry, it cannot be carbonized completely, resulting in inconvenient and time-consuming operation and much smoke.

*Pollen Typha* (PT) is a well-known TCM drug widely used in clinical for bleeding, antithrombosis (Akkol et al. 2011; Yan et al. 2017). Both the raw drug and the carbonized PT are listed in the CP (2015 edition). There are two methods to preparing charcoal PT. One is stir-frying them to deep yellow, and the other is baking them to brown (Chen et al. 2006). Compounds in the different process of products showed significant differences due to the heating time (Chen et al. 2015). Researchers found that after being heated for more than 7 min, the pollen grain were shrunken and broken, the colour turned to black, the contents of flavonoids glycosides decreased during the heating and their aglycones increased until after heated for 7 min. However, the mechanism of chemical composition transformation and haemostatic effect are still unclear. Furthermore, there are no strict control measures and corresponding quality standard for carbonized *Pollen Typhae* during the processing (Gao et al. 2019).

### Improved method

So far, new carbonizing methods have been developed: heating with sand, microwave carbonizing, calcining with muffle furnace and carbonizing with a drying oven, for examples. Carbonizing by sand has high heat conductivity, low cost, easy production and other features. High heat of sand can carbonize medicine and uneven heating of medicine can be avoided. This method can be used for the preparation of most charcoal drugs, such as carbonized *Nodus Nelumbinis Rhizomatis*, carbonized *Sanguisorba officinalis*, carbonized *Furctus Crataegi* and so on, but is not suitable for the preparation of light, fragile or non-separable drugs. For example, it is demonstrated that the haemostatic effects of four processing methods including raw, fried carbon, sand ironed, baked are very effective, and the best processing method of *Folium Artemisiae Argyi* is sand ironed, which is the shortest for blood coagulation (Zeng et al. 2011).

The principle of microwave processing is to realize strenuous motion and mutual friction of pharmaceutical molecules through microwave (Wu, Zhang et al. 2018). Comparing to the typical methods, microwave processing has obvious advantages. It is simple to operate and produces high quality charcoal drugs. In addition, such method gives advantages of high-speed processing in high precision, low pollution and has a wide prospect of application. It is suitable for the charcoal medicinal materials with light texture. With this approach, medicine was put in glassware and carbonized into externally brown and internally burned black products with high heat. Tan et al. (2019) reported that charred *Sophorae Fructus* can be processed by this method. The microwave processing technique of charred *Sophorae Fructus* with uniform appearance and controllable quality has simple operation, energy saving and high efficiency, which can be popularized in production (Tan et al. 2019). Carbonizing by baking can solve the problem of uncontrollable temperature and time. Researchers compared the haemostasis effect of *Sanguisorbae Radix* and charred *Sanguisorbae Radix*. The results showed that both have different haemostasis mechanisms, the function of haemostasis was more effective after charcoal by baking (Zhang et al. 2017).

## Basic research on haemostasis material in charcoal drugs

In terms of the haemostasis mechanism of charcoal drugs, TCM holds that ‘red stops when it meets black’. Most charcoal drugs have an effect of stopping blood. According to modern research, the haemostasis mechanism of charcoal drugs is highly complicated, which is not usually driven by a single factor. Primarily, the haemostasis mechanism promotes blood coagulation by strengthening coagulation factors or inhibiting anticoagulant factors in human bodies. Modern pharmacology has discussed the haemostasis mechanism of charcoal drugs by studying influences of TCM ingredients and effective parts before and after carbonizing on the blood coagulation system of animals and focuses on basic changes of material, such as carbon of TCM after carbonizing, dissolution of coagulant substances, reduction of anticoagulant substances and new water-soluble carbon dots (CDs).

### Traditional theory of bleeding stopping of charcoal drugs

Ancient people thought that red belongs to fire and black belongs to water. According to theory of the five elements, Water extinguishes Fire. In other words, black overcomes red. During the Yuan Dynasty, Ge Kejiu mentioned that, generally speaking, warm blood circulates, cold blood stagnates and bleeding stops when it meets black in ShiyaoShenshu. During the Ming Dynasty, Li Shizhen proposed all carbonized black medicines can be used to stop bleeding. During the Qing Dynasty, Wang Ang wrote, if drugs, such as *Gardenia jasminoides*, *Rhizoma Zingiberis*, *Sanguisorba officinalis*, *Trachycarpi Petiolus* and *Trogopterus Dung*, are used to stop bleeding, they should turn black by stir-frying to overcome red in BencaoBeiyao. Throughout history, many TCM scholars applied charcoal drugs to treatment of bleeding symptoms on the basis of the theory, leading to good effects. Shihuisan is a representative prescription for haemostasis and consist of 10 types of carbonized drugs, which is usually used to treat various bleeding symptoms caused by blood-heat bleeding.

## Modern research on bleeding stopping of charcoal drugs

### Theory of carbon

According to the theory, after carbonizing by high-temperature, TCM decoction pieces generate lots of carbon (the main component of activated carbon). From the perspective of TCM, due to strengthening of the astringent property of traditional Chinese medicine after carbonizing, adsorption and astringency effects of the medicine are strengthened, according with the multi-hole course structure of carbon and requiring further study. High-temperature carbonizing generates many loose holes in charcoal drugs, which can cause physical adsorption to accelerate haemostasis. In addition, due to special structure of carbon surface, it can activate plasma clotting factors and split blood platelet, releasing blood platelet factors, to promote coagulation. Meanwhile, it can release material strengthening tension of smooth muscles, causing vasoconstriction in favour of haemostasis. On the principle of ‘property maintenance by carbonizing’, during carbonizing, properties of TCM are maintained to some extent. Hence, a charcoal drug can be regarded as a combination of TCM and activated carbon. For example, the absorption capacity of carbon of carbonized *Radix Rehmanniae* and carbonized *Scutellaria Radix* are obviously increased, compared with corresponding raw medicines (Huang et al. 2013; Wang et al. 2013).

### Increase of procoagulant material

Tannin is a type of phenolic compound with an effect of convergence, which is the fourth most abundant sustainable biopolymers from plants, present in soft tissues of woody plants like bark, needles and leaves (Garcia et al. 2016). Tannins are commonly divided into two types: hydrolysable tannins and condensed tannins. Condensed tannins are widely distributed in nature and constitute more than 90% of commercial tannins of the total world production (Arbenz and Averous 2015). Due to their high chemical reactivity, tannins are widely used in thermosetting systems for many years, such as tannin-based foam material (Pizzi 2016), tannin-based adhesives for wood bonding and can be used as a reinforcement of polypropylene and UV protection properties (Bele et al. 2010; Samper et al. 2013; Liao, Brosse et al. 2019). It is generally considered that tannin increases after carbonizing and the main material basis for the haemostatic effect of charcoal drugs. Tannins are of high heat resistance. During carbonizing of some drugs, other constituents in cells can be transformed into Tannins. For example, after carbonizing of *Flos Sophorae*, their glycoside is condensed into tannins, which is converted from rutin. Both tannin and rutin could lower the capillary permeability (CP), the bleeding time (BT) and coagulation time (CT) in mice and also decrease the prothrombin time (PT) of plasma (Li et al. 2004). Tannin can contract capillaries of wounds and transform exudative protein into macromolecule precipitation that can attach itself to mucous membrane to stop bleeding. In some carbonized drugs, tannin, especially catechin and gallic acid, increase obviously, so the pharmacologic action is greatly enhanced. However, after carbonizing of some drugs, the content of tannins is reduced. Therefore, the content of tannins can provide theoretical support for the carbonizing haemostatic effect of some drugs, but it is not a rule appropriate for all charcoal drugs.

Calcium ions exist in most herbal TCM. Some studies have shown that the high temperature of carbonizing promotes ionization of calcium and release of soluble calcium ions, where Ca^2+^ increases obviously. It can promote protein coagulation in blood and activate numerous factors for polymerization of fibrin and blood coagulation and reduce permeability of capillaries and cell membranes. It is a cofactor of blood coagulation and regarded as a main basis for haemostasis. Microelements take part in synthesis of hormones and vitamins in human and animal bodies, which is regarded as the most important carrier and electronic transfer system (Xu and Xu 2009). It also regulates the level of free radicals and activates numerous biological enzymes in human bodies. However, some microelements, such as Pb, Hg and As, are harmful to human bodies. Some scholars hold, carbonizing has an influence on types and dissolution of microelements. Reasonable processing can reduce dissolution of harmful microelements and increase dissolution of beneficial microelements, leading to effect enhancement and toxicity reduction. *Rhizoma Coptidis* (Huanglian) is among the more commonly used herbal drugs in TCM, with the reported efficacy of suppressing fever, dispelling dampness, removing toxicosis and detoxification (Hung et al. 2018; Li et al. 2019; Zhu et al. 2019). Zhang XD found the concentration of elements in *Rhizoma Coptidis* under different processing methods, such as steaming, stir-frying and carbonizing. Eleven elements including As, Ca, Cd, Cu, Fe, Hg, K, Mg, Pb, Se and Zn were analysed by means of ICP-AES technique. Contents of these elements in the different processed samples were found at different levels. This suggest that these elements may play a direct or indirect role in the pharmacological effect or adverse effect of different processed *Rhizoma Coptidis* (Zhang et al. 2012). According to research, calcium ions increased significantly in some drugs after carbonizing. However, the current research cannot completely verify that the haemostatic effect of charcoal drugs is enhanced with the increase of calcium ions.

### Conversion of effective components of drugs

It is believed that carbonizing causes change of chemical components relating to haemostasis. During carbonizing, a high temperature can damage volatile oil with effects of promoting blood circulation and anticoagulating in drugs. In addition, it can generate or increase of anticoagulant components. For example, during carbonizing of *Sophora japonica*, the content of rutin reduces constantly, but quercetin increases. The haemostatic effect of quercetin is closely related to change of active constituents in the process of carbonizing (Zhu and Li 2018). In a study on contents of saccharic acid in cockscombs before and after carbonizing, it was found that contents of saccharic acid in 10 groups of carbonizd cockscombs were higher. However, no saccharic acid was detected before carbonizing. Hence, the glucoside was generated by transformation caused by high heat (Bao et al. 2011). Ginger is an important TCM with the reported efficacy of arthritis, rheumatism, sprains, muscular aches, pains, sore throats, cramps, constipation, indigestion, vomiting, hypertension, dementia, fever, infectious diseases and helminthiasis (Aktan et al. 2006; Al-Amin et al. 2006; Amin and Hamza 2006). Different processing methods can produce different processed gingers with dissimilar chemical constituents and pharmacological activities (Ali et al. 2008; Marx et al. 2013).

It is demonstrated that antioxidant activity of dried ginger was the highest, for its phenolic contents are 5.2-, 1.1- and 2.4-fold higher than that of fresh, stir-frying and carbonized ginger, respectively, the antioxidant activities' results indicated a similar tendency with phenolic contents: dried ginger > stir-frying ginger > fresh ginger > carbonized ginger. The processing contributed to the decreased concentration of gingerols and the increased levels of shogaols, which reducing the antioxidant effects in pace with processing (Li et al. 2016).

### Theory of carbon dots

Nanometer materials, such as graphene quantum dots and fluorescent semiconductor have attracted the interest of many researchers for drug delivery, cellular imaging high emitting diodes, and so on (Drummen 2010; Tang et al. 2012; Qiu et al. 2015). A CD usually refers to zero-dimensional carbon nanomaterial less than 10 nm, where its fluorescence emission wavelength relies on excitation wavelength (Baker and Baker 2010). Its main elements are C, H, O and N. Compared to traditional semiconductor quantum dots, CDS are superior in terms of high aqueous solubility, low toxicity, excellent biocompatibility and resistance to photo-bleaching (Li, Kang et al. 2011; Wang et al. 2011; Li, Kang et al. 2012). It is applied to multiple fields, such as biological imaging, cell labelling and tumour diagnosis and treatment (Yang et al. 2018; Fang et al. 2019; Gharat et al. 2019; Shaikh et al. 2019). Methods of CD synthesis include: chemical ablation (Ray et al. 2009); electrochemical carbonization (Shinde and Pillai 2013); laser ablation (Yang et al. 2009); microwave (Jaiswal et al. 2012); water/solvent thermal treatment; and high-temperature dissociation (Tang et al. 2019). After preparation, CDs separate nanometer materials of different size according to properties and differences of nano-particles by ultrafiltration, membrane filtration, AGE, column chromatography and gradient centrifugation. Since the discovery, CDs have attracted much attention and been constantly studied. People expect new CDs, especially CDs with bioactivity, that are economic, simple and green (Li, Zhao et al. 2012; Barman and Patra 2018). The high-temperature dissociation is similar to (high-temperature) carbonizing of charcoal drugs. Through research on nano-drugs, it was found that different charcoal drugs had different pharmacological activity (Abbasi et al. 2016).

**Figure 3.**
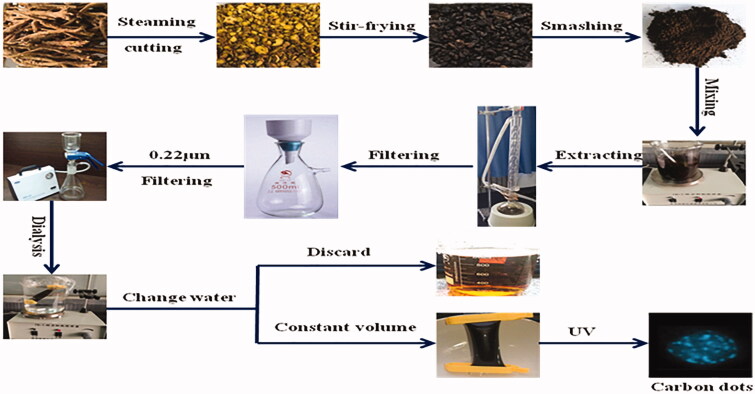
The preparation process of carbon dots of *Scutellaria baicalensis*.

Some scholars thought high-temperature carbonization was the key to generating a material basis for efficacy of charcoal drugs. In recent years, scholars introduced the characterization technique of the nanometer discipline into research on charcoal drugs, took clinically common charcoal drugs as objects of study and studied the material basis for the haemostatic effect of charcoal drugs (Lu et al. 2013). As a result, they found that CDs existed in many charcoal drugs and structural characteristics, physicochemical properties and biological activities of CDs in different charcoal drugs were different (Sun et al. 2018). By research on CDs in charcoal drugs, such as carbonized *Schizonepetae Herba*, carbonized *Cirsii Herba* and carbonized *Typhae Pollen*, it was found that CDs existed in all charcoal drugs and were characterized by high water-solubility, good biocompatibility and low toxicity. In addition, all extracted pure CDs showed a good effect of haemostasis (Luo et al. 2018; Zhang et al. 2018; Wang, Kong et al. 2018). *Schizonepetae Herba* carbonisata has been used in TCM to treat haemorrhagic diseases for more than 100 years, but little information is available on its haemostatic components and mechanism. CDs in charcoal drugs could enhance solubility of glycoside in water by affecting glycuronide. Some scholars studied influences of new water-soluble CDs on baicalin and found that pure CDs could significantly enhance solubility of baicalin in water. By comparison with icELISA, it was found that the oral bioavailability of the combination of CDs and baicalin was 1.7 times higher than that of pure baicalin (Luo et al. 2019). Through the preliminary experimental research, we found the similar CDs in the process of charring of *Scutellaria baicalensis.* The preparation process of CDs of *Scutellaria baicalensis* is shown in Figure 3.

According to research, by carbonizing, baicalin and wogonoside in *Scutellariae Radix* were converted into baicalein and wogonin which could be absorbed easily with strong effect of haemostasis. In the process of high-temperature carbonizing, CDs were one of the key substances that play a role in stopping bleeding and could be directly applied to treatment of blood-heat bleeding symptoms. Meanwhile, it could promote absorption of glycoside, which strengthens the effect of haemostasis indirectly.

## Discussion

Though carbonizing of TCM has a history for thousands of years and charcoal drugs have been widely used in clinical application, there are still many problems. First, since the Yuan Dynasty, the theory that ‘bleeding stops when it meets black’ has had a profound influence on application of charcoal drugs. However, the theory can only explain the haemostatic effect of partial charcoal drugs and cannot state the processing mechanism with modern science clearly. Second, although a total of 25 carbonized decoction pieces have been adopted in the latest CP, a large number of carbonized herbs are not covered. The methods of processing vary significantly in different areas and most carbonized herbs were recorded in the local standards of different province. Even in the latest CP, the carbonizing method is not accurately described. For example, most carbonized decoction pieces judged the degree of processing by colour change, so it is difficult to control the procedure of processing in actual operation. Another challenge is the change of composition. With the development of novel concepts and techniques, great advances have been achieved, but most parts of processing remain unclear. After carbonizing, both properties and efficacy of TCM change greatly, where change paths of chemical composition are unknown. Research relating to the material basis for the haemostatic effect of partial charcoal drugs still focuses on changes of chemical composition (Jiang et al. 2010). However, due to complex composition of TCM, many active ingredients of TCM are unknown. According to analysis of active constituents of charcoal drugs in modern research, main opinions include: calcium ions play a role in stopping bleeding; tannins play a leading role in stopping bleeding by charcoal drugs; conversion of drug constituents is closely related to strengthening of its haemostatic effect; carbon generated by carbonizing is the main substance causing a haemostatic effect. However, all of these studies only account for part of the pharmacodynamic material basis of some charcoal drug. So far, there is no widely recognized unified conclusion on the pharmacodynamic material basis and the related research is still a long-term arduous task. Preliminary statement on the haemostatic effect of partial charcoal drugs creates TCM factors affecting application and development of charcoal drugs. Influences of carbonizing on the processing mechanism are very complicated and involve TCM chemistry, biochemistry and pharmacology, etc. In addition, chemical composition of few TCM has been worked out, increasing difficulty in discussing the mechanism of charcoal drugs.

Hence, to reveal the substance of the theory and provide a scientific basis for clinical application of charcoal drugs, researchers should find a new path and conduct in-depth research to lay a foundation for the theory of charcoal drugs by applying modern science and technology and using related experience for reference.
